# Discriminant validity, responsiveness and reliability of the arthritis-specific Work Productivity Survey assessing workplace and household productivity in patients with psoriatic arthritis

**DOI:** 10.1186/ar4602

**Published:** 2014-07-04

**Authors:** Jane T Osterhaus, Oana Purcaru

**Affiliations:** 1 Wasatch Health Outcomes, 2613 Silver Cloud Drive, Park City, UT 84060, USA; 2 UCB Pharma, Allee de la Recherche, 60 1070 Brussels, Belgium

## Abstract

**Introduction:**

The novel arthritis-specific Work Productivity Survey (WPS) was developed to estimate patient productivity limitations associated with arthritis within and outside the home, which is an unmet need in psoriatic arthritis (PsA). The WPS has been validated in rheumatoid arthritis. This report assesses the discriminant validity, responsiveness and reliability of the WPS in adult-onset PsA.

**Methods:**

Psychometric properties were assessed using data from the RAPID-PsA trial (NCT01087788) investigating certolizumab pegol (CZP) efficacy and safety in PsA. WPS was completed at baseline and every 4 weeks until Week 24. Validity was evaluated at baseline via known-groups defined using first and third quartiles of patients’ Disease Activity Score 28 based on C-reactive protein (DAS28(CRP)), Health Assessment Questionnaire-Disability Index (HAQ-DI), Short Form-36 (SF-36) items and PsA Quality of Life (PsAQoL) scores. Responsiveness and reliability were assessed by comparing WPS mean changes at Week 12 in American College of Rheumatology 20% improvement criteria (ACR20) or HAQ-DI Minimal Clinically Important Difference (MCID) 0.3 responders versus non-responders, as well as using standardized response means (SRM). All comparisons were conducted on the observed cases in the Randomized Set, regardless of the randomization group, using a non-parametric bootstrap-t method.

**Results:**

Compared with patients with a better health state, patients with a worse health state had on average 2 to 6 times more household work days lost, more days with reduced household productivity, more days missed of family/social/leisure activities, more days with outside help hired and a significantly higher interference of arthritis per month. Among employed patients, those with a worse health state had 2 to 4 times more workplace days lost, more days with patient workplace productivity reduced, and a significantly higher interference of arthritis on patient workplace productivity versus patients with a better health state. WPS was also responsive to clinical changes, with responders having significantly larger improvements at Week 12 in WPS scores versus non-responders. The effect sizes for changes in productivity in ACR20 or HAQ-DI MCID responders were moderate (0.5 < SRM < 0.8) or small.

**Conclusions:**

These analyses demonstrate the validity, responsiveness and reliability of the WPS, as an instrument for the measurement of patient productivity within and outside the home in an adult-onset PsA population.

## Introduction

Psoriatic arthritis (PsA) is an inflammatory arthritis that occurs in up to one-third of patients with psoriasis. In about 80% of PsA cases, the arthritis develops after the appearance of psoriasis [1]. The risk of a patient with psoriasis developing arthritis is greater in individuals with severe psoriasis, yet occasionally severe arthritis may occur with minimal skin disease.

While there is published evidence for the burden of the disease on work disability and the validity of different measurement tools in related rheumatic diseases such as rheumatoid arthritis (RA) and ankylosing spondylitis [2], to date there are limited data on work disability in PsA [3,4]. Some evidence indicates that the employment rate in patients with PsA is significantly lower than that seen in the general population and is slightly lower than in the ankylosing spondylitis population, but is higher than in the RA population [5].

In contrast to RA, very little is known about the indirect costs associated with PsA and its treatment. Only three published studies assessing indirect monetary costs associated with PsA investigated the cost of presenteeism, which is expected to be one of the larger components of the indirect costs of PsA. One study only assessed indirect costs in terms of costs due to short-term absence from work and permanent work disability [6]. The remaining two studies only assessed indirect costs in terms of costs due to short-term absence [7,8]; the second of these studies included costs due to both caregivers’ and patients’ short-term absences from work [8].

The lack of information describing the indirect costs of PsA does not reflect its impact on society. The age of onset of PsA is generally between 30 and 50 years [9,10], with the mean age of diagnosis estimated at 41 to 44 years of age [9,11], at which stage many men and women are in the midst of their working careers. Additionally, two separate studies to identify issues of concern amongst PsA patients reported that reduced ability to work or volunteer [12], to fulfill personal roles, and to participate in social life were common concerns [13]. Further research to understand the impact of PsA and its treatment on patient work productivity is therefore warranted.

At a PsA workshop held during the Outcome Measures in Rheumatology (OMERACT) 7 meeting, participants discussed domains that should be included in PsA clinical trials, and generated a final list of aspects to be included, which was presented and ratified; 61% of respondents indicated that participation (in activities such as housework and remunerative employment) should be included as a domain, compared with 48% for fatigue and 91% for physical function [14]. The core set of domains, for inclusion in all clinical trials of PsA, included joints, skin, patient’s global assessment of disease activity, pain, function, and health-related quality of life (HRQoL). The domain of participation was considered potentially important but still in need of research regarding its inclusion and how to assess it [15].

In order to fully quantify the impact of an intervention on patient productivity, it is crucial to consider the entire patient productivity continuum both within the work and home environments [16]. Historically, there has been an unmet need for an instrument designed to assess presenteeism and absenteeism in both the workplace and household. This has been highlighted in several reviews assessing the properties of self-reported instruments measuring principally worker productivity [17-20].

The arthritis-specific Work Productivity Survey (WPS) was developed to fulfill the unmet need for an arthritis-specific instrument to assess the impact of an intervention on patient productivity within the work and home environments, in addition to daily activities during the preceding month [21]. The WPS has demonstrated properties of discriminative validity, reliability and responsiveness for the measurement of patient productivity within and outside the home in patients with active RA [21].

There is no gold standard measure for assessing productivity in PsA. During the OMERACT 9 meeting, based on the available filter evidence (truth, discrimination and feasibility) the WPS was one of six instruments identified by the OMERACT Worker Productivity Group as a possible candidate for assessing worker productivity changes in rheumatology [22].

The WPS was selected to estimate patient productivity limitations associated with PsA because of the ease of use and positive response, in terms of psychometric properties, seen in RA [21], and the similarity in terms of work disability associated with RA and PsA.

The objective of this paper was to assess the discriminant validity, responsiveness and reliability of the WPS in adult-onset PsA.

## Materials and methods

### Patients and study design

The psychometric validation of the WPS was conducted using data from the double-blind period of the RAPID-PsA study (efficacy and safety of certolizumab pegol (CZP) in PsA), which was double blind and placebo controlled to week 24, dose blind to week 48 and then open label to week 216 [23]. The first 24 weeks of the RAPID-PsA study investigated CZP 200 mg every 2 weeks, 400 mg every 4 weeks or placebo, and was conducted at 92 sites across North America, Latin America, Western Europe and Central/Eastern Europe from March 2010 to November 2011. Institutional review boards or ethics committees approved the protocol at each center (see Additional file 1). All patients gave written informed consent, and the study was conducted in accordance with the Declaration of Helsinki [23].

Patients were randomly assigned 1:1:1 to receive placebo (saline) every 2 weeks or subcutaneous CZP 400 mg at weeks 0, 2 and 4 (loading dose) followed by either CZP 200 mg every 2 weeks or CZP 400 mg every 4 weeks until week 24 [23]. Placebo patients who failed to achieve a 10% improvement from baseline in both swollen and tender joints at weeks 14 and 16 were eligible for blinded mandatory escape to active treatment. These patients were re-randomized to active treatment at week 16 in a 1:1 ratio (CZP 200 mg every 2 weeks:CZP 400 mg every 4 weeks), receiving loading doses at weeks 16, 18 and 20. CZP patients continued to receive the initially assigned dose [23].

The primary efficacy endpoint was the American College of Rheumatology 20% improvement criteria (ACR20) response at week 12 [24,25]. Secondary endpoints included the American College of Rheumatology 50% improvement criteria (ACR50), the American College of Rheumatology 70% improvement criteria (ACR70), Disease Activity Score – 28-joint count based on C-reactive protein (DAS28(CRP)), physical functioning as measured by the Health Assessment Questionnaire – Disability Index (HAQ-DI) and the Psoriatic Arthritis Response Criteria (PsARC), psoriatic skin involvement as measured by the Psoriasis Area and Severity Index (PASI), HRQoL assessed using the Short Form-36 health survey (SF-36), EuroQoL-5 dimensions (EQ-5D), Psoriatic Arthritis Quality of Life (PsAQoL) and Dermatology Life Quality Index (DLQI), and productivity measured using the WPS.

### Questionnaires

The WPS is a disease-specific questionnaire assessing the impact of arthritis on patient workplace and household productivity and on daily activities during the preceding month. The questionnaire is interviewer administered, self-reported by the patient and has a 1-month recall period [21]. Full details on its development have been reported previously [21].

The first item of the WPS addresses current labor market participation (‘are you currently employed outside the home?’), which is a strong indicator of work ability since not working implies complete loss of paid productivity. Normative and comparative data on employment status are also assessed. Two items capture self-reported absenteeism (days of work missed) and presenteeism (days with patient productivity reduced by at least one-half) due to arthritis, and two items capture the same concepts but applied to nonpaid (household) work. Additional items capture the respondent’s estimate of the extent to which arthritis has interfered with the patient’s work productivity (paid and nonpaid) on a scale of 0 to 10 (0 = no interference and 10 = complete interference), the number of days in the last month that outside help was hired because of arthritis, and the number of days in the last month that family, social or leisure activities were missed because of arthritis [21].

The HAQ-DI is a patient-reported questionnaire that provides an assessment of the impact of the disease on ability to perform activities of daily living [26]. HAQ-DI scores of 0 to 1 generally represent mild to moderate functional difficulty, scores of 1 to 2 represent moderate to severe functional difficulty, and scores of 2 to 3 indicate severe to very severe limitations of physical function or disability [27]. In this study, the interpretation of within-patient changes used for the HAQ-DI scores was based on a minimum clinically important difference (MCID) of 0.3 points [28]; this threshold was used to define responders.

The DAS28(CRP) is a composite score measuring disease activity, with lower scores indicating lower disease activity [29].

The PASI is a measure of severity of psoriatic skin involvement [30,31]. The PASI ranges from 0 (no disease) to 72 (maximal disease). The PASI75 response is based on at least 75% improvement from baseline in the PASI score. In RAPID-PsA, the PASI and PASI75 response were assessed in the subgroup of patients with psoriasis involving at least 3% of body surface area at baseline.

A PsARC response is defined as at least a 30% improvement of tender joint count or swollen joint count, as well as a one-point improvement on a five-point scale in the Patient’s Global Assessment of Disease Activity and the Physician’s Global Assessment of Disease Activity, and no worsening of any of these scores [32].

The PsAQoL questionnaire was developed by McKenna and colleagues as a quality-of-life measure specific to adult patients with PsA [13]. The PsAQoL global score ranges from 0 to 20, with a lower score indicating a better HRQoL.

The DLQI, developed in 1994, was the first dermatology-specific quality-of-life instrument [33]. The DLQI score ranges from 0 to 30, where higher scores represent greater impairment on the quality of life.

The SF-36 is a widely used generic HRQoL instrument that evaluates eight health domains: physical functioning, role physical, bodily pain, general health, vitality, social functioning, role emotional, and mental health [34]. The eight domains are summarized in two component summaries: the physical component summary (PCS) and the mental component summary (MCS). Scores for the SF-36 range between 0 and 100, with higher scores indicating a better HRQoL.

The EQ-5D questionnaire is comprised of a five-item health status measure and a visual analog scale (VAS) [35,36]. Each of the five dimensions is divided into three levels – no problem, some or moderate problems, and extreme problems – and is scored as 1, 2, and 3, respectively. The EQ-5D VAS records the respondent’s self-rated health status on a vertical 20 cm scale, graduated from 0 to 100 (0 = worst imaginable health status, 100 = best imaginable health status).

The ACR20/ACR50/ACR70 response assesses the treatment of symptoms and signs in patients with active PsA. A patient is defined as an ACR20/ACR50/ACR70 responder if there is an improvement (that is, reduction) of at least 20%/50%/70%, respectively, from baseline in the tender joint count, swollen joint count and at least three of the five core set measures: Patient’s Assessment of Arthritis Pain (VAS), Patient’s Global Assessment of Disease Activity (VAS), Physician’s Global Assessment of Disease Activity (VAS), an acute-phase reactant (C-reactive protein), and physical functioning based on the HAQ-DI [24].

Further details of the questionnaires assessed have been included in Additional file 2.

### Data handling and statistical analysis

The assessment of the psychometric properties (discriminant validity, responsiveness and reliability) of the WPS was performed on the observed cases in the overall randomized set (RS) population (that is, all patients randomized into the study), regardless of the randomization group.

### Discriminant validity

Given the nature of the WPS questionnaire – which is composed of several single–global questions scored and interpreted separately – and the length of the recall period of these questions, the construct validity of the WPS questionnaire was evaluated by means of discriminant validity, using correlations and the known-groups validation method [37]. The association between the responses to WPS Questions 2 to 9 and scores of the different measures assessing the disease activity, physical functioning or HRQoL was assessed through Kendall correlation coefficients. Given the diverse nature of the measures tested, and the current lack of or limited research into patient productivity in PsA, *a priori* hypotheses were made regarding the degree to which different outcomes correlate with the WPS.

Given the difference between the concepts assessed by the WPS questions and the other measures considered, the *a priori* hypothesis tested was that the correlation coefficients are expected to be low to moderate (low, 0 to 0.3; moderate, ≥0.3 to <0.5), thus indicating a divergent validity of the measures compared [37]. High correlations could imply a low discriminant validity and suggest that two items are measuring similar concepts [37]. The Kendall association coefficients were evaluated between WPS Questions 2 to 9 and the following selected measures: DAS28(CRP), PASI (in patients with at least 3% body surface area at baseline), HAQ-DI, SF-36 MCS, PCS and domains, PsAQoL, DLQI and EQ-5D VAS.

The known-groups validity method was used to compare the patient productivity scores between patients with a worse health state and patients with a better health state. Patients with a higher disease activity, worse HRQoL or lower physical functioning were considered to be in a worse health state, whereas patients with lower disease activity, better HRQoL or higher physical functioning were considered to be in a better health state. The hypothesis tested through the known-groups validity method was that patients with a worse health state were expected *a priori* to have higher losses in productivity at work outside home and within the household (that is, higher WPS scores for Questions 2 to 9) due to their disease compared with patients with a better health state. The known-groups were formed using the first and third quartile scores for each outcome as cutoff points. Patients were considered to have better HRQoL if they had baseline SF-36 scores ≥ third quartile or a PsAQoL or DLQI baseline score ≤ first quartile, whereas those with SF-36 scores ≤ first quartile or PsAQoL or DLQI scores ≥ third quartile were defined as having worse HRQoL. Similarly, better physical functioning was defined as HAQ-DI ≤ first quartile or SF-36 physical functioning domain (or PCS scores) ≥ third quartile, with worse physical functioning delineated by HAQ-DI ≥ third quartile and SF-36 ≤ first quartile. Patients with DAS28(CRP) or PASI score ≤ first quartile were considered to have low disease activity/severity, whereas DAS28(CRP) or PASI score ≥ third quartile indicated high disease activity/severity.

The discriminant validity of the WPS was assessed using baseline observed data. To test the validity of patient productivity at paid work (WPS Questions 2 to 4), cutoff points were computed using only patients employed outside the home; whereas for productivity within the home (Questions 5 to 9), the thresholds were computed using all patients. Sensitivity analyses were performed using a median cutoff threshold.

A nonparametric bootstrap *t* method was used to compare the mean WPS question responses between the known-groups [38]. This method was favored because of the highly skewed distribution of the WPS scores. Bootstrap analyses were performed with 10,000 replications. A variance-stabilizing transformation was used in order to adjust for dependence between the bootstrap values and the corresponding standard error.

### Responsiveness to clinical changes and reliability

The responsiveness of the WPS to clinical changes in a patient’s condition over time was evaluated by comparing the changes from baseline in productivity scores between clinical responders versus nonresponders at week 12 (as measured by ACR20). The hypothesis tested was that clinical responders were expected *a priori* to have higher improvements in productivity at work outside home and within the household versus nonresponders, reflected by higher negative changes (in absolute value) in WPS scores.

According to the primary analysis, a patient was considered a responder if they met the criteria of ACR20 improvement over baseline at week 12. Any patient who did not meet the ACR20 or who withdrew from the study for any reason before week 12 was considered a nonresponder.

The reliability of the WPS was tested in conjunction with the responsiveness to the ACR20 clinical response by comparing the changes in WPS scores in patients achieving ACR50, ACR70, PASI75 (in patients with at least 3% body surface area at baseline), HAQ-DI MCID, PsARC and DAS28(CRP) remission (DAS28(CRP) < 2.6) responses at week 12 versus nonresponders.

WPS score changes from baseline at week 12 were compared between week 12 clinical responders and nonresponders using a nonparametric bootstrap *t* method. A variance-stabilizing transformation was used in order to adjust for dependence observed between bootstrap values and the corresponding standard error. Bootstrap analyses were performed with 10,000 replications.

In addition to the comparison of the changes in WPS scores between the clinical responders and nonresponders, the standardized response mean (SRM) was calculated. The SRM is one of the most widely used measures of the effect size of the response, indicating whether the change was large relative to the variability of the measurements. The SRM is estimated as the mean change in scores between two visits divided by the standard deviation of that change in scores. Thresholds for the SRM (absolute values) were proposed by Cohen and colleagues in order to interpret the size of the effects: small, from 0.2 to 0.5; moderate, from 0.5 to 0.8; and large, >0.8 [39].

The responsiveness and reliability of the WPS was assessed at week 12 in all RS patients, regardless of the randomization group.

## Results

### Population characteristics

A total of 409 patients were randomized and 368 (90%) patients completed the 24-week phase.

Overall, RS patients had a mean age of approximately 48 years (range 19 to 75 years), slightly more than one-half (55.3%) of the patients were female and almost all patients were white (97.8%). The mean body mass index overall was nearly 30 kg/m^2^, with 40.3% of patients having body mass index ≥30 kg/m^2^ (Table 1).

**Table 1:** Baseline demographics and clinical characteristics (randomized set, observed cases)

	**All randomized patients (*n*** **= 409)**
Demographic characteristics	
Age (years)	47.6 ± 11.4
Sex (% female)	55.3
Race (% white)	97.8
Weight (kg)	84.4 ± 18.8
Body mass index (kg/m^2^)	29.8 ± 6.5
Arthritis characteristics	
Time from psoriatic arthritis diagnosis^a^ (years)	8.6 ± 8.2
C-reactive protein^b^ (mg/l)	8.0 (0.1 to 238.0)
Mean tender joint count (0 to 68 joints)	20.3 ± 14.9
Mean swollen joint count (0 to 66 joints)	10.7 ± 8.0
Physician’s Assessment of Disease Activity, by VAS (mm)	57.9 ± 18.6
Patient’s Assessment of Disease Activity	59.1 ± 20.7
Patient’s Assessment of Arthritis Pain, by VAS (mm)	60.3 ± 20.4
DAS28 (CRP)	5.0 ± 1.0
Mean HAQ-DI (range 0 to 3)	1.31 ± 0.64
Psoriasis characteristics	
Psoriasis BSA ≥3% (%)	61.6
PASI^c^	11.7 ± 11.9
Prior use of synthetic DMARDs (%)	50.6
**1**	47.2
**≥2**	
Prior TNF inhibitor exposure (%)	19.6
Health-related quality of life	
Mean SF-36 MCS	41.7 ± 12.1
Mean SF-36 PCS	33.4 ± 7.7
Mean PsAQoL	11.1 ± 5.6
Mean DLQI	8.5 ± 7.2
Mean EQ-5D VAS	49.9 ± 20.4

Data presented as mean ± SD, percentage or median (minimum to maximum). BSA, body surface area; DAS28(CRP), Disease Activity Score – 28-joint count based on C-reactive protein; DLQI, Dermatology Life Quality Index; DMARD, disease-modifying antirheumatic drug; EQ-5D, EuroQoL-5 dimensions; HAQ-DI, Health Assessment Questionnaire – Disability Index; MCS, mental component summary; PASI, Psoriasis Area and Severity Index; PCS, physical component summary; PsAQoL, Psoriatic Arthritis Quality of Life; SF-36, Short Form-36 items; TNF, tumor necrosis factor; VAS, visual analogue scale. ^a^From the start date of the primary disease. ^b^Normal range < 8.0 mg/l. ^c^PASI scores are presented for patients with psoriasis body surface area ≥ 3% at baseline.

The mean duration of PsA for all patients was 8.6 years, and 80.0% of patients had disease duration of at least 2 years. Allowed disease-modifying antirheumatic drugs at baseline and prior tumor necrosis factor inhibitor treatment were equally distributed. Approximately 20% of patients had received previous treatment with tumor necrosis factor inhibitors. The mean C-reactive protein for all patients was 15.9 mg/l; small differences in mean C-reactive protein values across groups were not clinically meaningful. The mean HAQ-DI and DAS28(CRP) scores were similar across treatment groups (1.31 and 5.01 points overall, respectively) and were indicative of moderate disease activity.

By geographic region, the largest percentage of patients came from Central/Eastern Europe (47.9% overall). Distribution by region was equally distributed across treatment groups.

At baseline, 59.4% of all patients were employed outside the home, 14% were unable to work due to PsA and 13.5% were retired; the rest were homemakers (6.1%), unable to work due to non-PsA health problems (3.4%), students (1.2%) or had other unemployment status (2.2%) (Table 2).

**Table 2:** **Patient employment status**^**a **^**at baseline (randomized set, observed cases)**

	**All randomized patients (*n*** **= 409)**
Employment status	
Employed outside the home	243 (59.4)
Homemakers	25 (6.1)
Retired	55 (13.5)
Students	5 (1.2)
Unable to work due to arthritis	57 (14.0)
Unable to work due to nonarthritis health problems	14 (3.4)
Other	9 (2.2)
Job function if employed	
Nonmanual	111 (27.3)
Manual with no supervisory duties	89 (21.9)
Mixed (manual and nonmanual)	42 (10.3)

^a^Captured through the Work Productivity Survey. Data presented as *n* (%).

### Baseline productivity within and outside the home

The burden of PsA at study baseline was high. Within the workplace, the mean number of days of work missed was 2.0 over the previous month, while the mean number of days per month with patient productivity reduced by at least one-half was 4.7. The impact of PsA on household productivity and participation in daily activities was even higher than within the workplace (Table 3).

**Table 3:** Workplace and household productivity at baseline (randomized set, observed cases)

	**All randomized patients (*n*** **= 409)**		
**WPS question**^**a**^	***n***	**Mean (SD)**	**Median**
2. Number of days of work missed (absenteeism)^b^	243	2.0 (5.33)	0.0
3. Number of days with productivity ≤50% at work (presenteeism)^b,c^	243	4.7 (7.41)	0.0
4. Rate of arthritis interference with work productivity^b,d^	242	4.1 (2.83)	5.0
5. Number of days of household work missed	406	5.7 (8.41)	2.0
6. Number of days with productivity ≤50% in household work^c^	406	7.6 (8.67)	5.0
7. Number of days of family, social or leisure activities missed	406	3.7 (7.25)	0.0
8. Number of days with outside help	409	2.4 (6.43)	0.0
9. Rate of arthritis interference with household work productivity^d^	409	5.0 (2.95)	5.0

SD, standard deviation. ^a^Recall period for Work Productivity Survey (WPS) is 1 month. ^b^Assessed in employed patients only. ^c^Days counted exclude those counted in previous question. ^d^Scored using numeric rating scale of 0 to 10, where 0 = no interference and 10 = complete interference.

Patients in jobs with some manual component had a similar number of workplace days missed per month to those in exclusively nonmanual jobs (mean 2.1 vs. 2.0 days). However, these patients reported more days per month with patient workplace productivity reduced by at least one-half compared with those with exclusively nonmanual jobs (mean 5.7 vs. 3.6 days respectively). Patient bias may play a part in these results, because it is probable that patients with more severe PsA symptoms would be less likely to take on manual roles. In terms of household work, employed patients reported less impact of PsA symptoms compared with nonemployed patients and with patients unable to work due to arthritis (mean household work days missed/month: 4.0 vs. 8.1 vs. 12.8; mean days per month with household productivity reduced by ≥50%: 6.3 vs. 9.5 vs. 13.5, respectively).

### Completion rates of WPS at baseline

At baseline, all patients in the RS population answered at least one of the WPS questions; there were no patients with a completely missing WPS questionnaire. The completion rates of each of the WPS questions at baseline in the RS population were very high, indicating that the instrument was clear, acceptable and representative of the study population, and therefore that the results can be generalizable to a larger PsA population. There were only three (0.7%) missing answers to WPS Questions 5 to 9 at baseline. Among all employed RS patients at baseline who were required to answer WPS Questions 2 to 4, the completion rates were also high, with only one (0.4%) missing answer (for Question 4).

At baseline, there was no presence of a ceiling effect as shown by the very small number of patients with a maximal answer. In the RS population, three (1.2%) and eight (3.3%) of the employed patients had an answer ≥30 days for WPS Question 2 and Question 3 respectively, and nine (3.7%) had a maximum answer of 10 for Question 4. Among all patients, 10 to 28 (2.5 to 6.9%) patients had an answer ≥30 days for WPS Questions 5 to 8 or a maximal score of 10 for Question 9.

As expected, in terms of floor effect, the percentage of patients with a minimal response varied between the different WPS questions, with a higher number of patients answering 0 for Question 2 (work days missed in the past month, 73.7% out of the employed RS population) and for Question 8 (days with outside help hired, 77.3% out of the entire RS population), and ranging from 14.3 to 57.6% for the other questions.

### Discriminant validity

The first hypothesis – tested using the association coefficients between WPS Questions 2 to 9 and different continuous measures assessing the disease activity, physical functioning and HRQoL – was confirmed. Coefficients were low (<0.3) to moderate (≥0.3 to 0.5) as expected, indicating divergent validity between the individual WPS questions and the other measures considered (Table 4).

**Table 4:** Kendall association coefficients between WPS and clinical and HRQoL assessments at baseline (randomized set, observed cases)

	**WPS question**							
	**2: number of work days missed because of arthritis**	**3: number of days with work productivity reduced by one-half or more because of arthritis**	**4: rate of arthritis interference with work productivity on a scale of 0 to 10**	**5: number of days with no household work because of arthritis**	**6: number of days with household work productivity reduced by one-half or more because of arthritis**	**7: number of days missed of family, social, or leisure activities because of arthritis**	**8: number of days with outside help hired because of arthritis**	**9: rate of arthritis interference with household work productivity on a scale of 0 to 10**
DAS28 (CRP)	0.16**	0.09	0.16**	0.22**	0.13**	0.15**	0.12*	0.24**
PASI^a^	*0.11*	0.06	−0.02	−0.01	0.05	0	0.02	−0.04
HAQ-DI	0.25**	0.21**	0.32**	0.34**	0.26**	0.28**	0.22**	0.38**
SF-36 MCS	−0.16**	−0.16**	−0.22**	−0.23**	−0.24**	−0.34**	−0.24**	−0.28**
SF-36 PCS	−0.19**	−0.15**	−0.28**	−0.31**	−0.20**	−0.19**	−0.15**	−0.32**
SF-36 PF	−0.22**	−0.16**	−0.27**	−0.36**	−0.22**	−0.25**	−0.20**	−0.34**
SF-36 BP	−0.29**	−0.20**	−0.33**	−0.34**	−0.24**	−0.33**	−0.19**	−0.38**
SF-36 RE	−0.24**	−0.21**	−0.28**	−0.29**	−0.23**	−0.35**	−0.30**	−0.31**
SF-36 RP	−0.26**	−0.24**	−0.34**	−0.32**	−0.26**	−0.28**	−0.23**	−0.37**
SF-36 GH	−0.07	−0.09	−0.18**	−0.16**	−0.15**	−0.15**	−0.17**	−0.20**
SF-36 MH	−0.15*	−0.15*	−0.20**	−0.19**	−0.23**	−0.29**	−0.21**	−0.27**
SF-36 SF	−0.17**	−0.21**	−0.26**	−0.30**	−0.26**	−0.36**	−0.17**	−0.30**
SF-36 VT	−0.14*	−0.10*	−0.18**	−0.27**	−0.22**	−0.27**	−0.17**	−0.30**
PsAQoL	0.24**	0.23**	0.30**	0.28**	0.28**	0.37**	0.27**	0.35**
DLQI	*0.11*^#^	0.03	0.06	0.08^#^	0.09*	0.15**	0.08^#^	0.10*
EQ-5D VAS	−0.09	−0.09	−0.19**	−0.15**	−0.13**	−0.14**	−0.12**	−0.20**

^a^Assessed only in patients with at least 3% body surface area at baseline. **P ≤ 0.001. *P ≤ 0.01. ^#^*P* ≤ 0.05.

WPS Questions 2 to 4 were assessed in employed patients only, whereas Questions 5 to 9 were assessed in all patients; Questions 4 and 9 are 0 to 10 scales, where 0 = no interference and 10 = complete interference. Correlation level (absolute value): low correlations, 0 to 0.1 (plain text), 0.1 (inclusive) to 0.3 (italic); moderate correlation, ≥0.3 to <0.5 (bold). HAQ-DI, lower score = better; DAS28, lower score = better; PASI, lower score = better; PsAQoL, lower score = better; DLQI, lower score = better; SF-36, higher score = better; EQ-5D VAS, higher score = better. BP, bodily pain; DAS28(CRP), Disease Activity Score – 28-joint count based on C-reactive protein; DLQI, Dermatology Life Quality Index; DMARD, disease-modifying antirheumatic drug; EQ-5D, EuroQoL-5 dimensions; GH, general health; HAQ-DI, Health Assessment Questionnaire – Disability Index; HRQoL, health-related quality of life; MCS, mental component summary; MH, mental health; PASI, Psoriasis Area and Severity Index; PCS, physical component summary; PsAQoL, Psoriatic Arthritis Quality of Life; PF, physical functioning; RE, role emotional; RP, role physical; SF, social functioning; SF-36, Short Form-36 items; VAS, visual analogue scale; VT, vitality; WPS, Work Productivity Survey.

The level and the sign (positive or negative) of the Kendall association coefficients indicated that better patient productivity at work and within home (that is, lower answers to WPS Questions 2 to 9) was associated with better HRQoL, better physical activity and lower disease activity (Table 4). Although the association coefficients between patient productivity within and outside the home and HRQoL were low to moderate, the association coefficients versus DLQI were very low, indicating that increased patient productivity levels within and outside the home were not associated with the DLQI level. Very low positive or negative association coefficients were also observed between PASI and the WPS questions, indicating no association between these two measures (Table 4).

The second hypothesis regarding validity, tested using known-groups analyses, was also confirmed, with results indicating that there was a higher burden of arthritis on patient productivity at both paid work and within the home in patients with a worse health state versus patients with a better health state. Among employed patients in the RS population, patients with a worse health state reported two to four times more work days lost, more work days with patient productivity reduced by one-half, and a higher interference of arthritis on work productivity compared with patients with a better health state (all *P* < 0.05 except for days with productivity reduced by 50% versus DAS28(CRP)) (Table 5).

**Table 5:** WPS baseline scores assessed by defined known-groups: patient workplace productivity (randomized set, observed cases)

**Instrument**^**a**^	**Number of days of work missed over the previous month**		**Number of days with productivity ≤50% at work over the previous month**		**Rate of arthritis interference with work productivity over previous month**^**b**^	
	**Worse**	**Better**	**Worse**	**Better**	**Worse**	**Better**
HAQ-DI	3.51*	0.99	6.77*	3.17	5.71**	3.00
(cutoff 0.75 and 1.50)	*n* = 71	*n* = 78	*n* = 71	*n* = 78	*n* = 71	*n* = 78
DAS28(CRP)	3.54**	0.57	5.30	3.10	4.49^#^	3.18
(cutoff 4.13 and 5.64)	*n* = 61	*n* = 61	*n* = 61	*n* = 61	*n* = 61	*n* = 61
SF-36 PCS	3.18*	0.87	6.85*	2.51	5.61**	2.69
(cutoff 29.56 and 40.43)	*n* = 61	*n* = 61	*n* = 61	*n* = 61	*n* = 61	*n* = 61
SF-36 MCS	2.89**	0.33	7.20**	3.03	5.32**	3.15
(cutoff 35.77 and 52.45)	*n* = 61	*n* = 61	*n* = 61	*n* = 61	*n* = 61	*n* = 61
PsAQoL	3.67**	0.47	8.11**	1.85	5.47**	2.71
(cutoff 6.00 and 15.00)	*n* = 61	*n* = 78	*n* = 61	*n* = 78	*n* = 61	*n* = 78
PASI	2.79	1.33	4.49	3.25	3.67	3.78
(cutoff 3.60 and 14.40)	*n* = 39	*n* = 40	*n* = 39	*n* = 40	*n* = 39	*n* = 40

Data presented as mean. DAS28(CRP), Disease Activity Score – 28-joint count based on C-reactive protein; HAQ-DI, Health Assessment Questionnaire – Disability Index; MCS, mental component summary; PASI, Psoriasis Area and Severity Index; PCS, physical component summary; PsAQoL, Psoriatic Arthritis Quality of Life; SF-36, Short Form-36 items; WPS, Work Productivity Survey. ^a^Cutoff points represent the first and third quartiles of baseline scores. Worse state defined for each individual measure as: DAS28(CRP) score ≥ third quartile, HAQ-DI ≥ third quartile, PsAQoL ≥ third quartile, SF-36 MCS ≤ first quartile, SF-36 PCS ≤ first quartile, PASI ≥ third quartile. Better state defined for each individual measure as: DAS28(CRP) score ≤ first quartile, HAQ-DI ≤ first quartile, PsAQoL ≤ first quartile, SF-36 MCS ≥ third quartile, SF-36 PCS ≥ third quartile, PASI ≤ first quartile. ^b^WPS Question 4 is a 0 to 10 scale, where 0 = no interference and 10 = complete interference. **P ≤ 0.001. *P ≤ 0.01. ^#^*P* ≤ 0.05. Nonparametric bootstrap *t* method with a variance stabilizing transformation, 10,000 replications.

At baseline, employed patients with a worse HRQoL, assessed through the SF-36 MCS or PCS score, had lost on average three times more full days of paid work per month (mean 2.9 or 3.2 days respectively) compared with patients with a better HRQoL (mean 0.3 or 0.9 days respectively) (Table 5). In terms of days with reduced patient productivity at work, employed patients with a worse HRQoL as assessed by SF-36 MCS or PCS had a significantly higher number of workdays with reduced patient productivity (mean 7.2 and 6.9 days per month, respectively) compared with patients with a better HRQoL (mean 3.0 and 2.5 days, respectively) (Table 5). Similar differences were observed in patients with a worse versus better PsA-specific HRQoL as assessed by PsAQoL (Table 5). Similar significant differences were also observed when comparing the rate of arthritis interference with patient work productivity in employed patients with a better HRQoL compared with patients with a worse HRQoL (Table 5).

Employed patients with worse physical functioning, as assessed by HAQ-DI or SF-36 PCS score, had a significantly higher number of work days lost and of days with reduced patient productivity per month, as well as a higher rate of arthritis interference with patient work productivity when compared with patients with better physical functioning (Table 5). Similarly, employed patients with a lower disease activity, as assessed by a DAS28(CRP) score ≤ first quartile, had a significantly lower number of work days lost per month, as well as a lower rate of arthritis interference with patient work productivity when compared with patients with higher disease activity (Table 5).

Similar patterns were observed when examining the differences in household productivity, as assessed by the WPS (Table 6). Compared with patients with a better health state, patients with a worse health state reported on average two to six times more days lost of household work, more days with household productivity reduced by at least one-half, more days missed of family, social or leisure activities, more days with outside help hired and a higher interference of arthritis per month (Table 6).

**Table 6:** WPS baseline scores assessed by defined known-groups: household productivity and daily activities (randomized set, observed cases)

**Instrument**^**a**^	**Number of days of household work missed over the previous month**		**Number of days with household productivity ≤50% at work over the previous month**		**Number of days of missed family, social or leisure activities over the previous month**		**Number of days with outside help over the previous month**		**Rate of arthritis interference with household work productivity over previous month**^**b**^	
	**Worse**	**Better**	**Worse**	**Better**	**Worse**	**Better**	**Worse**	**Better**	**Worse**	**Better**
HAQ-DI	9.97**	1.60	11.97*	3.74	7.28**	1.43	4.82**	0.67	6.75**	3.22
(cutoff 0.88 and 1.75)	*n* = 116	*n* = 116	*n* = 116	*n* = 116	*n* = 116	*n* = 116	*n* = 116	*n* = 116	*n* = 116	*n* = 116
DAS28 (CRP)	8.44**	2.70	9.26**	5.60	6.00**	1.58	3.68*	1.07	6.11**	3.70
(cutoff 4.23 and 5.73)	*n* = 103	*n* = 101	*n* = 103	*n* = 101	*n* = 103	*n* = 101	*n* = 103	*n* = 101	*n* = 103	*n* = 101
SF-36 PCS	11.09**	1.37	10.81**	4.22	7.74**	1.37	4.65**	0.63	6.54**	3.30
(cutoff 27.99 and 38.28)	*n* = 101	*n* = 98	*n* = 101	*n* = 98	*n* = 101	*n* = 98	*n* = 101	*n* = 98	*n* = 101	*n* = 98
SF-36 MCS	10.10**	2.50	11.25**	5.19	7.91**	0.40	5.34**	0.18	6.65**	3.54
(cutoff 32.76 and 50.78)	*n* = 100	*n* = 101	*n* = 100	*n* = 101	*n* = 100	*n* = 101	*n* = 100	*n* = 101	*n* = 100	*n* = 101
PsAQoL	9.16**	1.70	11.44**	2.94	7.74**	0.35	4.97**	0.12	6.75**	3.04
(cutoff 6.00 and 16.00)	*n* = 104	*n* = 103	*n* = 104	*n* = 103	*n* = 104	*n* = 103	*n* = 104	*n* = 103	*n* = 104	*n* = 103
PASI	4.69	6.21	8.18	5.68	3.08	4.35	1.81	2.21	4.40	4.89
(cutoff 3.70 and 15.50)	*n* = 62	*n* = 66	*n* = 62	*n* = 66	*n* = 62	*n* = 66	*n* = 62	*n* = 66	*n* = 62	*n* = 66

Data presented as mean. DAS28(CRP), Disease Activity Score – 28-joint count based on C-reactive protein; HAQ-DI, Health Assessment Questionnaire – Disability Index; MCS, mental component summary; PASI, Psoriasis Area and Severity Index; PCS, physical component summary; PsAQoL, Psoriatic Arthritis Quality of Life; SF-36, Short Form-36 items; WPS, Work Productivity Survey. ^a^Cutoff points represent the first and third quartiles of baseline scores. Worse state defined for each individual measure as: DAS28(CRP) score ≥ third quartile, HAQ-DI ≥ third quartile, PsAQoL ≥ third quartile, SF-36 MCS ≤ first quartile, SF-36 PCS ≤ first quartile, PASI ≥ third quartile. Better state defined for each individual measure as: DAS28(CRP) score ≤ first quartile, HAQ-DI ≤ first quartile, PsAQoL ≤ first quartile, SF-36 MCS ≥ third quartile, SF-36 PCS ≥ third quartile, PASI ≤ first quartile. ^b^WPS Question 4 is a 0 to 10 scale, where 0 = no interference and 10 = complete interference. **P ≤ 0.001. *P ≤ 0.01. ^#^*P* ≤ 0.05. Nonparametric bootstrap *t* method with a variance stabilizing transformation, 10,000 replications.

Compared with patients with a worse HRQoL, patients with a better HRQoL (as assessed by SF-36 PCS, MCS and PsAQoL) missed significantly fewer days of household work (1.4 to 2.5 vs. 9.2 to 11.1 mean days per month, *P* <0.05), had significantly fewer days with household work productivity reduced by at least one-half due to arthritis (2.9 to 5.2 vs. 10.8 to 11.4 mean days), had lost a significantly lower number of days of family, social or leisure activities (0.4 to 1.4 vs. 7.7 to 7.9 mean days) and had significantly fewer days with outside help hired due to arthritis over the preceding month (0.1 to 0.6 vs. 4.7 to 5.3 mean days) (Table 6). The rate of arthritis interference on household work productivity reported by patients with a worse HRQoL was significantly higher compared with patients with a better HRQoL (on average 3.0 to 3.5 vs. 6.5 to 6.8 on a 0-point to 10-point scale) (Table 6). Significant differences were also obtained when the known-groups were defined using the SF-36 domains (data not shown).

Consistent findings were observed in patients with better versus worse physical functioning as measured by the HAQ-DI and SF-36 PCS, as well as in patients with a lower versus higher disease activity (Table 6).

The known-groups analysis defined based on PASI were inconclusive, as no consistent or significant differences were seen for any of the WPS questions (Tables 5 and 6). These findings are in line with the association analysis, where the Kendall correlation coefficient between the PASI and WPS questions did not indicate that the skin index is associated with productivity scores within or outside the home.

Sensitivity analyses using median cutoff values (see Additional file 3) demonstrated similar trends, with patients in worse health states reporting poorer worker productivity outcomes compared with patients in better health states. Slight numerical differences of the sensitivity analysis compared with the base analysis may be due to the fact that patients within the outlying quartiles are likely to have more extreme health states.

### Responsiveness and reliability

#### WPS changes from baseline by ACR20 response at week 12

**Figure 1:**
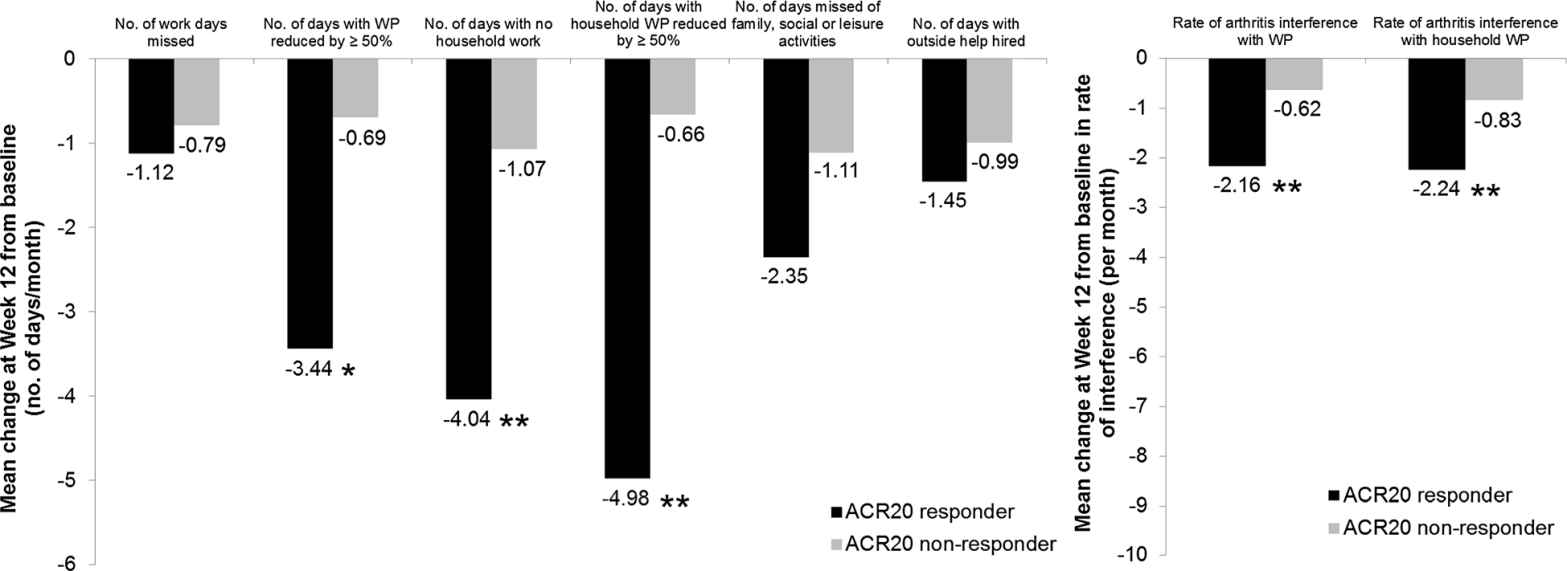
**Mean changes from baseline in the Work Productivity Survey by American College of Rheumatology 20% improvement criteria clinical response at week 12.** Change from baseline in the Work Productivity Survey by American College of Rheumatology 20% improvement criteria (ACR20) clinical response at week 12 (randomized set, observed cases). **P ≤ 0.001, *P < 0.01 responders versus nonresponders; *P*-values were obtained using the nonparametric bootstrap *t* method. Rate of interference is a score on a scale of 0 to 10 points (0 = no interference and 10 = complete interference). WP, work productivity.

**Figure 2:**
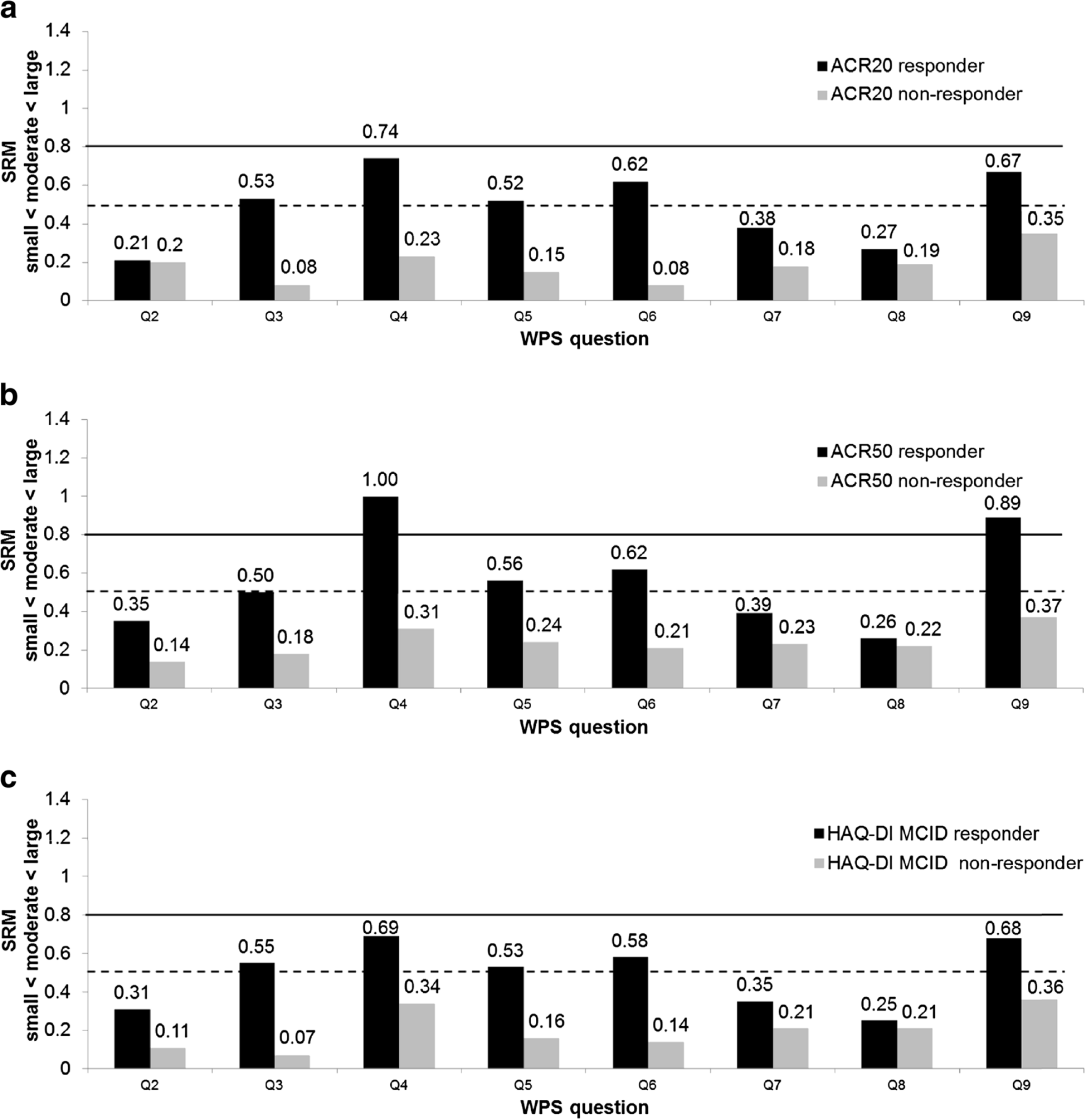
**Effect size of mean changes from baseline in the Work Productivity Survey at week 12 (randomized set, observed cases).** Effect size (standardized response mean (SRM)) of mean changes from baseline in the Work Productivity Survey (WPS) are presented by the **(a)** American College of Rheumatology 20% improvement criteria (ACR20) clinical response, **(b)** American College of Rheumatology 50% improvement criteria (ACR50) clinical response, and **(c)** Health Assessment Questionnaire – Disability Index (HAQ-DI) minimal clinically important difference (MCID of 0.3) clinical response. SRM (absolute values) thresholds: small, from 0.2 to 0.5 (below the dashed line); moderate, from 0.5 to 0.8 (between the two lines); and large, >0.8 (above the solid line).

The responsiveness hypothesis was confirmed, with significantly greater improvements in household and patient workplace productivity observed in ACR20 responders versus nonresponders at week 12 (except in work days missed (WPS Question 2) and days with outside help (WPS Question 8), where only numerical differences were seen) (Figure 1).The effect sizes of the changes in productivity scores, measured by the SRM, were negligible (SRM < 0.1) or small (SRM < 0.5) in ACR20 nonresponders; whereas moderate effect sizes were observed in ACR20 responders (or small in the case of days missed at work (WPS Question 2), days missed of family/social/leisure activities (WPS Question 7) and days with outside help hired (WPS Question 8)) (Figure 2a).

#### WPS changes from baseline by other response measures at week 12

With regards to the reliability hypothesis, differences observed in HAQ-DI MCID and ACR50 responders versus nonresponders were similar and consistent with those reported for ACR20. When assessing the responsiveness of WPS to more stringent clinical responses, like ACR50, the effect sizes in mean changes in patient productivity within and outside the home in the ACR50 responder group were moderate to large (or small in the case of days missed at work (WPS Question 2), days missed of family/social/leisure activities (WPS Question 7) and days with outside help hired (WPS Question 8)) (Figure 2b). Effect sizes observed when assessing the WPS responsiveness in HAQ-DI MCID responders versus nonresponders were also similar to those observed for ACR20 (Figure 2c). For the PsARC response, the effect sizes in mean changes in patient productivity scores were low in all WPS questions except Question 4 (rate of interference with work productivity) and Question 9 (rate of interference with household work productivity), where the effect sizes were moderate.

The results based on the ACR70 and Disease Activity Score – 28-joint count remission criteria were inconclusive due to a large imbalance in the sample sizes of the two groups compared. Although the effect sizes of the mean changes in WPS responses compared with the PASI75 response were small to moderate, there were no statistically significant differences in changes in productivity scores between PASI75 responders and nonresponders.

## Discussion

The disease-specific WPS is a tool developed to estimate patient productivity limitations due to arthritis in the workplace and in household activities [21], whose psychometric properties have already been demonstrated in subjects with active RA [21]. Previous work demonstrated that the WPS could efficiently evaluate both the burden of the disease and clinical interventions on work outcomes in patients with RA [21,40,41].

The objective of this paper was to evaluate the initial psychometric properties of WPS in an adult-onset PsA population. The discriminant validity, the responsiveness to clinical changes and the reliability of the survey were therefore evaluated in subjects enrolled in a clinical trial for the treatment of active PsA.

OMERACT 6 and OMERACT 7 have highlighted the importance to patients of considering the impact of arthritic conditions on paid and unpaid work outcomes [42,43], as they represent an important component of the health and wellbeing of rheumatology patients. Similar thinking should apply to patients with PsA. Patient-reported outcomes have been included in rheumatology trials because they capture the patient’s perspective of the disease process and the impact of treatments on the disease. The impact of PsA on work outcomes is not currently a core component of rheumatology clinical trials; however, it is an endpoint of interest to both patients and employers. OMERACT has re-enforced the importance of work productivity as an outcome measure in rheumatology through the Worker Productivity Special Interest Group, which has reviewed specific productivity instruments and continues to evaluate concepts and methodological and interpretation issues surrounding work productivity [44].

The findings indicated that the WPS instrument was generally well understood by patients, as indicated by the high completion rates. As in RA, the WPS demonstrated good discriminant validity both in terms of association coefficients and known-groups analyses evaluated against a range of different continuous measures assessing disease activity (DAS28(CRP)), physical functioning (HAQ-DI, SF-36 PCS and SF-36 physical functioning), and HRQoL (SF-36, PsAQoL, EQ-5D). The Kendall association coefficients indicated the divergent validity between the individual WPS questions and these measures, which were further confirmed by the known-groups analyses. Compared with patients in a better health state, patients in a worse health state had on average two to six times greater patient productivity losses due to PsA within the household. Similar findings were observed among employed patients, with patients in a worse health state reporting on average two to four times greater patient productivity losses within the workplace compared with patients with a better health state.

The known-groups were constructed using the first and third quartiles of the instrument scores at baseline in order to avoid comparison of unbalanced groups [21]. These analyses were further confirmed using a median cutoff of the score (see Additional file 3). The responsiveness of the WPS was assessed using clinically recognized thresholds, and supports the discriminant validity analysis.

The WPS was responsive to clinical changes, as measured by the ACR20 response. Clinical responders at week 12 had significantly larger improvements in patient productivity within and outside the home and in daily activities compared with nonresponders. Similar findings were observed when using a different clinical response (for example, HAQ-DI MCID, ACR50), supporting the responsiveness and the reliability of the WPS. The observed differences in WPS score changes and the effect sizes seen for clinical responders and nonresponders, and the similarities in the responsiveness of the WPS questions when using different response criteria, support the responsiveness and reliability of the WPS in patients with PsA.

WPS was not found to be associated with the skin-related measures DLQI and PASI response. This may indicate that these aspects of disease have relatively little impact on the productivity of patients with PsA.

Among all WPS questions, the work days missed due to PsA and the days with outside help hired did show a certain level of responsiveness, but not as large or consistent as the other WPS questions. The number of respondents who reported full days missed or days with outside help hired was quite small relative to the entire study sample. This suggests that in this sample PsA interfered with patient work productivity, but was less likely to lead to full disability. This also suggests that PsA patients might not necessarily hire outside help, but does not exclude the possibility of receiving help from relatives or friends. Given the utility of WPS across a variety of rheumatic conditions, including those where higher levels of disability would be anticipated, all questions of the WPS should remain, ensuring an accurate assessment of the impact of arthritis on different aspects of patient work and household productivity.

The limitations of this study included the patient population, which was recruited for a clinical trial of active PsA and may not be completely representative of the wider PsA population. However, as the WPS is currently developed as a tool for clinical trials, the patient population used should provide sufficient evidence to ensure the validity of this measure in its current role. The WPS is also affected by the normal limitations of self-reported questionnaires, although previous reports have confirmed that self-reporting is still the best means of collecting this type of data [45].

In the absence of a gold standard to measure patient work productivity in randomized clinical trials, the criterion validity of the WPS could not been assessed. Instead, another type of validity (that is, discriminant) has been evaluated in the current paper. A cross-cultural validation of the WPS has not yet been assessed, given the objective and quantitative nature of the WPS. Work to further support the use of this questionnaire to assess workplace and household productivity is ongoing.

## Conclusions

The present analysis is the first report on the psychometric properties of the WPS in PsA, demonstrating its validity, responsiveness and reliability. This survey can be used to capture the impact of active PsA on patient productivity in the workplace and within the household, and participation in daily activities. It is also a credible instrument for use in clinical trials and in clinical practice to assess the impact of treatment on PsA-related patient workplace and household productivity losses.

## Abbreviations

ACR20: American College of Rheumatology 20% improvement criteria; ACR50: American College of Rheumatology 50% improvement criteria; ACR70: American College of Rheumatology 70% improvement criteria; CZP: certolizumab pegol; DAS28(CRP): Disease Activity Score – 28-joint count based on C-reactive protein; DLQI: Dermatology Life Quality Index; EQ-5D: EuroQoL-5 dimensions; HAQ-DI: Health Assessment Questionnaire – Disability Index; HRQoL: health-related quality of life; MCID: minimal clinically important difference; MCS: mental component summary; OMERACT: Outcome Measures in Rheumatology; PASI: Psoriasis Area and Severity Index; PCS: physical component summary; PsA: psoriatic arthritis; PsAQoL: Psoriatic Arthritis Quality of Life; PsARC: Psoriatic Arthritis Response Criteria; RA: rheumatoid arthritis; RS: randomized set; SF-36: Short Form-36 items; SRM: standardized response mean; VAS: visual analog scale; WPS: Work Productivity Survey.

## Competing interests

JTO is a paid consultant for UCB S.A. (Belgium) OP is employed full-time by UCB Pharma (Brussels, Belgium).

## Authors’ contributions

JTO made substantial contributions to the conceptualization, analysis and interpretation of the data. OP participated in the conceptualization, analysis and interpretation of the data. Both authors reviewed and approved the manuscript.

## Supplementary Material

Additional file 1 is a table presenting a list of ethical bodies approving the RAPID-PsA study. Click here for file

Additional file 2 is a table presenting details of the questionnaires assessed in the article. Click here for file

Additional file 3 **is Tables S1 and S2 presenting WPS baseline scores assessed by defined known-groups.** Known-groups analysis using median scores as the cutoff for analysis. Click here for file
