# Supplementary material for: Discriminant validity, responsiveness and reliability of the arthritis-specific Work Productivity Survey assessing workplace and household productivity in patients with psoriatic arthritis
Source: Arthritis Res Ther. 2014 Jul 4;16(4):R140. doi: 10.1186/ar4602 (PMC4226958; doi:10.1186/ar4602)
Supplement: Additional file 1 — is a table presenting a list of ethical bodies approving the RAPID-PsA study. [file ar4602-S1.docx]

List of Institutional Review Boards/Independent Ethics Committees

| Site number | IRB/IEC name, address, and committee chairperson |
| --- | --- |
| 100 | St. Vincent’s Healthcare Limited  Ethics and Medical Research Committee  Elm Park  Dublin 4  IRELAND  Chairperson: Dr. B. Kirby |
| Central IEC  Belgium | Commissie Medische Ethiek  UZ Leuven campus Gasthuisberg  Herestraat 49  3000 Leuven  BELGIUM  Chairperson: Prof. W. Van den Bogaert, MD |
| 150 | See above; Commissie Medische Ethiek UZ Leuven campus Gasthuisberg |
| 151 | See above; Commissie Medische Ethiek UZ Leuven campus Gasthuisberg |
| 152 | See above; Commissie Medische Ethiek UZ Leuven campus Gasthuisberg |
| 153 | See above; Commissie Medische Ethiek UZ Leuven campus Gasthuisberg |
| Central IEC  France | Comité de Protection des Personnes (CPP)  Ile de France VIII  Hôspital Ambrose Paré  Laboratoire d’Anatomopathologie  9, avenue Charles-de-Gaulle  92100 Boulogne-Billancourt  FRANCE  Chairperson: Frédérique Barthod, MD |
| 201 | See above; Comité de Protection des Personnes (CPP) Ile de France VIII |
| 202 | See above; Comité de Protection des Personnes (CPP) Ile de France VIII |
| 203 | See above; Comité de Protection des Personnes (CPP) Ile de France VIII |
| 204 | See above; Comité de Protection des Personnes (CPP) Ile de France VIII |
| 205 | See above; Comité de Protection des Personnes (CPP) Ile de France VIII |
| 206 | See above; Comité de Protection des Personnes (CPP) Ile de France VIII |
| Central IEC  Germany | Ethik-Kommission der Ärztekammer Westfalen-Lippe und der Medizinischen Fakultät der Westfälischen Wilhelms-Universität Münster  Von-Esmarch-Str. 62  48149 Münster  GERMANY  Chairperson: Prof. Dr. Hans-Werner Bothe, MA |
| Central IEC  Germany | Ethik-Kommission des Fachbereichs Medizin der Johann Wolfgang Goethe-Universität Frankfurt  Theodor-Stern-Kai 7  60590 Frankfurt am Main  GERMANY  Chairperson: Prof. Dr. med. Sebastian Harder |
| Central IEC  Germany | Ethik-Kommission des Landes Berlin  Fehrbelliner Platz 1  10707 Berlin  GERMANY  Chairperson: PD Dr. med. K. Thomas Moesta |
| Central IEC  Germany | Ethik-Kommission der Medizinischen Fakultät der  Ludwig-Maximilians Universität, München  Pettenkoferstr. 8a  80336 München  GERMANY  Chairperson: Prof. Dr. Wolfgang Eisenmenger |
| 250 | See above; Ethik-Kommission der Ärztekammer Westfalen-Lippe und der Medizinischen Fakultät der Westfälischen Wilhelms-Universität Münster |
| 252 | See above; Ethik-Kommission der Ärztekammer Westfalen-Lippe und der Medizinischen Fakultät der Westfälischen Wilhelms-Universität Münster;  See above; Ethik-Kommission des Fachbereichs Medizin der Johann Wolfgang Goethe-Universität Frankfurt |
| 253 | See above; Ethik-Kommission der Ärztekammer Westfalen-Lippe und der Medizinischen Fakultät der Westfälischen Wilhelms-Universität Münster;  Ethikkommission bei der Sächsischen Landesärztekammer  Schützenhöhe 16  01099 Dresden  GERMANY  Chairperson: Prof. Dr. med. Rolf Haupt |
| 254 | See above; Ethik-Kommission der Ärztekammer Westfalen-Lippe und der Medizinischen Fakultät der Westfälischen Wilhelms-Universität Münster;  Ethik-Kommission der Ärztekammer Hamburg  Humboldtstr. 67 a  22083 Hamburg  GERMANY  Chairperson: Prof. Dr. med. Thomas Weber |
| 255 | See above; Ethik-Kommission der Ärztekammer Westfalen-Lippe und der Medizinischen Fakultät der Westfälischen Wilhelms-Universität Münster;  Ethik-Kommission der Albert-Ludwigs-Universität Freiburg  Engelberger Str. 1  79106 Freiburg  GERMANY  Chairperson: Prof. Dr. Drs. Hc Stefan Pollak |
| 256 | See above; Ethik-Kommission der Ärztekammer Westfalen-Lippe und der Medizinischen Fakultät der Westfälischen Wilhelms-Universität Münster;  Ethik-Kommission der Ärztekammer Nordrhein  Tersteegenstr. 31  40474 Düsseldorf  GERMANY  Chairperson: Prof. Dr. Ursula Sehrt-Ricken (Essen) |
| 257 | See above; Ethik-Kommission der Ärztekammer Westfalen-Lippe und der Medizinischen Fakultät der Westfälischen Wilhelms-Universität Münster;  See above; Ethik-Kommission des Landes Berlin |
| 258 | See above; Ethik-Kommission der Ärztekammer Westfalen-Lippe und der Medizinischen Fakultät der Westfälischen Wilhelms-Universität Münster;  See above; Ethik-Kommission des Landes Berlin |
| 259 | See above; Ethik-Kommission der Ärztekammer Westfalen-Lippe und der Medizinischen Fakultät der Westfälischen Wilhelms-Universität Münster;  Ethik-Kommission der Ärztekammer Niedersachsen  Ethik-Kommission zur Beurteilung medizinischer Forschung am Menschen  Berliner Allee 20  30175 Hannover  GERMANY  Chairperson: Prof. Dr. Peter Kleine, Dr. jur. |
| 260 | See above; Ethik-Kommission der Ärztekammer Westfalen-Lippe und der Medizinischen Fakultät der Westfälischen Wilhelms-Universität Münster;  See above; Ethik-Kommission der Medizinischen Fakultät der Ludwig-Maximilians Universität, München |
| 262 | See above; Ethik-Kommission der Ärztekammer Westfalen-Lippe und der Medizinischen Fakultät der Westfälischen Wilhelms-Universität Münster;  See above; Ethik-Kommission des Fachbereichs Medizin der Johann Wolfgang Goethe-Universität Frankfurt |
| 263 | See above; Ethik-Kommission der Ärztekammer Westfalen-Lippe und der Medizinischen Fakultät der Westfälischen Wilhelms-Universität Münster;  See above; Ethik-Kommission der Medizinischen Fakultät der Ludwig-Maximilians Universität, München |
| Central IEC  Hungary | Egészségügyi Tudományos Tanács Klinikai Farmakológiai Etikai Bizottsága  Arany János. u. 6-8  1051 Budapest  HUNGARY  Chairperson: Dr. Susanna Fürst |
| 300 | See above; Egészségügyi Tudományos Tanács Klinikai Farmakológiai Etikai Bizottsága |
| 301 | See above; Egészségügyi Tudományos Tanács Klinikai Farmakológiai Etikai Bizottsága |
| 302 | See above; Egészségügyi Tudományos Tanács Klinikai Farmakológiai Etikai Bizottsága |
| 303 | See above; Egészségügyi Tudományos Tanács Klinikai Farmakológiai Etikai Bizottsága |
| 304 | See above; Egészségügyi Tudományos Tanács Klinikai Farmakológiai Etikai Bizottsága |
| 306 | See above; Egészségügyi Tudományos Tanács Klinikai Farmakológiai Etikai Bizottsága |
| 350 | Comitato Etico per la Sperimentazione Clinica dei Medicinali dell’Azienda Ospedaliera Universitaria Pisani di Pisa  Via Roma, 67  56126 Pisa  ITALY  Chairperson: Prof. Romano Danesi |
| 351 | Comitato Etico per la Sperimentazione Clinica dei Medicinali dell’Azienda Ospedaliera-Universitaria Careggi  Largo Brambilla, 3  50134 Firenze  ITALY  Chairperson: Dr. ssa Raffaella Giardiello |
| 352 | Comitato Etico dell’Azienda Sanitaria Unica Regionale Delle Marche Di Ancona  Via Caduti del Lavoro, 40  60131 Ancona  ITALY  Chairperson: Dott. Gilberto Gentili |
| Central IEC  The Netherlands | Medische-Ethische Commissie academisch ziekenhuis Maastricht en Maastricht University (METC azM/UM)  P. Debyelaan 25  Postbus 5800  6202 AZ Maastricht  THE NETHERLANDS  Chairperson: Mw. Dr. CED de Die-Smulders |
| 400 | See above; Medische-Ethische Commissie academisch ziekenhuis Maastricht en Maastricht University (METC azM/UM) |
| 401 | See above; Medische-Ethische Commissie academisch ziekenhuis Maastricht en Maastricht University (METC azM/UM) |
| Central IEC  Poland | Komisja Bioetyczna przy Okregowej Izbie Lekarskiej w Krakowie  Krupnicza 11a  31-123 Krakow  POLAND  Chairperson: kol. Stefan Bednarz |
| 450 | See above; Komisja Bioetyczna przy Okregowej Izbie Lekarskiej w Krakowie |
| 452 | See above; Komisja Bioetyczna przy Okregowej Izbie Lekarskiej w Krakowie |
| 453 | See above; Komisja Bioetyczna przy Okregowej Izbie Lekarskiej w Krakowie |
| 454 | See above; Komisja Bioetyczna przy Okregowej Izbie Lekarskiej w Krakowie |
| 455 | See above; Komisja Bioetyczna przy Okregowej Izbie Lekarskiej w Krakowie |
| 456 | See above; Komisja Bioetyczna przy Okregowej Izbie Lekarskiej w Krakowie |
| 457 | See above; Komisja Bioetyczna przy Okregowej Izbie Lekarskiej w Krakowie |
| 458 | See above; Komisja Bioetyczna przy Okregowej Izbie Lekarskiej w Krakowie |
| 459 | See above; Komisja Bioetyczna przy Okregowej Izbie Lekarskiej w Krakowie |
| 462 | See above; Komisja Bioetyczna przy Okregowej Izbie Lekarskiej w Krakowie |
| 463 | See above; Komisja Bioetyczna przy Okregowej Izbie Lekarskiej w Krakowie |
| Central IEC  Czech Republic | Etická komise IKEM a FTNsP  Fakultní Thomayerova nemocnice s polikfinikou  Videnská 800  140 59 Praha 4 – Krc  CZECH REPUBLIC  Chairperson: Prof. MUDr. Vladimír Stanék, CSc. |
| Central IRC  Czech Republic | Etická komise  Revmatologický ústav  Na Slupi 4  128 50 Praha 2  CZECH REPUBLIC  Chairperson: RNDR. Ivana Pútová |
| 500 | See above; Etická komise IKEM a FTNsP, Fakultní Thomayerova nemocnice s polikfinikou;  See above; Etická komise Revmatologický ústav |
| 501 | See above; Etická komise IKEM a FTNsP, Fakultní Thomayerova nemocnice s polikfinikou;  See above; Etická komise Revmatologický ústav |
| 502 | See above; Etická komise IKEM a FTNsP, Fakultní Thomayerova nemocnice s polikfinikou;  See above; Etická komise Revmatologický ústav |
| 503 | See above; Etická komise IKEM a FTNsP, Fakultní Thomayerova nemocnice s polikfinikou;  See above; Etická komise Revmatologický ústav |
| 504 | See above; Etická komise IKEM a FTNsP, Fakultní Thomayerova nemocnice s polikfinikou;  See above; Etická komise Revmatologický ústav |
| 505 | See above; Etická komise IKEM a FTNsP, Fakultní Thomayerova nemocnice s polikfinikou;  See above; Etická komise Revmatologický ústav |
| Central IEC  Spain | Comité Ético de Investigación Clinica de Galicia  Unidad: Subdirección Xeral de Farmacia e Produtos Sanitarios. Conselleria de Sanidade  San Lázaro, s/n  15703 Santiago Compostela (La Coruña)  SPAIN  Chairperson: Dr. Rosendo Bugarín González |
| Central IEC  Spain | CEIC Autonómico de Ensayos  Clinicos de Andalucía  Conserjería de Salud  Avenida de la Innovación, s/n  Edificio Arena 1  41020 Sevilla  SPAIN  Chairperson: Dr. Demetrio Mariano Aguayo Canela |
| 550 | See above; Comité Ético de Investigación Clinica de Galicia  Unidad: Subdirección Xeral de Farmacia e Produtos  Sanitarios. Conselleria de Sanidade;  CEIC Hospital Universitario Infanta Cristina  Departamento de Farmacología de la Facultad de Medicina  Avenida de Elvas, s/n  06008 Badajoz  SPAIN  Chairperson: Dra. Francisca Lourdes Márquez Pérez |
| 551 | See above; Comité Ético de Investigación Clinica de Galicia  Unidad: Subdirección Xeral de Farmacia e Produtos  Sanitarios. Conselleria de Sanidade;  See above; CEIC Autonómico de Ensayos, Clinicos de Andalucía, Conserjería de Salud;  Secretaría Técnica del CEIC  Hospital Universitario Reina Sofia  Edificio Consultas Externas pl-1  Avenida Menéndez Pidal, s/n  14004 Córdoba  SPAIN  Chairperson: Dr. Jose Luis Barranco Quintana |
| 552 | See above; Comité Ético de Investigación Clinica de Galicia  Unidad: Subdirección Xeral de Farmacia e Produtos  Sanitarios. Conselleria de Sanidade |
| 553 | See above; Comité Ético de Investigación Clinica de Galicia  Unidad: Subdirección Xeral de Farmacia e Produtos  Sanitarios. Conselleria de Sanidade;  See above; CEIC Autonómico de Ensayos, Clinicos de Andalucía, Conserjería de Salud;  CEIC del Hospital Universitario Virgen Macarena  Avenida Dr. Fedriani, 3  2a planta, Unidad de Investigación  41009 Sevilla  SPAIN  Chairperson: Dr. D. Miguel Ángel Rico Corral |
| 555 | See above; Comité Ético de Investigación Clinica de Galicia  Unidad: Subdirección Xeral de Farmacia e Produtos  Sanitarios. Conselleria de Sanidade;  Secretaria Téchnica CEIC  Hospital Universitario de La Paz  Hospital General, Planta 8o  Despachos 818 y 819  Paseo de la Castellana, 261  28046 Madrid  SPAIN  Chairperson: Dr. Rosendo Bugarin González |
| Central IEC  United Kingdom | Leicestershire, Northamptonshire and Rutland  Research Ethics Committee 2  The Old Chapel  Royal Standard Place  Nottingham  NG1 6GN  UNITED KINGDOM  Chairperson: Dr. Carl Edwards |
| 600 | See above; Leicestershire, Northamptonshire and Rutland Research Ethics Committee 2;  Leeds Teaching Hospital NHS Trust  Research & Development Directorate  34 Hyde Terrace  Leeds  LS2 9LN  UNITED KINGDOM  Chairperson: Dr. Steve Smye |
| 601 | See above; Leicestershire, Northamptonshire and Rutland Research Ethics Committee 2;  Salford Royal NHS Foundation Trust  Research & Development Department  Summerfield House  554 Eccles New Road  Salford  M5 5AP  UNITED KINGDOM  Chairperson: Prof. Bill Ollier |
| 602 | See above; Leicestershire, Northamptonshire and Rutland Research Ethics Committee 2;  Whipps Cross University Hospital  Research & Development Unit  Room 24, Willow Lodge  Leytonstone  London  E11 1NR  UNITED KINGDOM  Chairperson: Mr. James Green |
| 603 | See above; Leicestershire, Northamptonshire and Rutland Research Ethics Committee 2;  West Suffolk Hospital NHS Trust  Research & Development Office  Bury St. Edmonds  IP33 2QZ  UNITED KINGDOM  Chairperson: Dr. John Hall |
| 604 | See above; Leicestershire, Northamptonshire and Rutland Research Ethics Committee 2;  Sheffield Teaching Hospital NHS Trust  Research & Development  1st Floor  11 Brommfield Road  Sheffield  S10 2SE  UNITED KINGDOM  Chairperson: Prof. Simon Heller |
| 605 | See above; Leicestershire, Northamptonshire and Rutland Research Ethics Committee 2;  Barnsley Hospital  Research & Development Directorate  Gawber Road  Barnsley  S75 2EP  UNITED KINGDOM  Chaiperson: Prof. Stuart G. Parker |
| Central IEC  Argentina | Comité Independiente de Ética para Ensayos en Farmacología Clínica  J. E. Uriburu 774, 1st Floor, Apt. C  (C1027AAP) Ciudad Autónoma de Buenos Aires  ARGENTINA  Chairperson: Prof. Dr. Luis M. Zieher |
| 700 | See above; Comité Independiente de Ética para Ensayos en Farmacología Clínica;  Comité de Docencia e Investigación de Consultorios Reumatológicos Pampa  La Pampa 1548, 1st Floor, Apt. A  (C1428DZF) Ciudad Autónoma de Buenos Aires  ARGENTINA  Chairperson: Dr. Alberto Rodriguez Velez |
| 702 | See above; Comité Independiente de Ética para Ensayos en Farmacología Clínica;  Comité de Docencia e Investigación  Centro Médico Privado de Reumatología  Lavalle 506  (T4000AXL) San Miguel de Tucumán  Tucumán  ARGENTINA  Chairperson: Prof. Dra. Sofía Amenabar |
| 704 | See above; Comité Independiente de Ética para Ensayos en Farmacología Clínica;  Comité de Docencia e Investigación  Organización Médica para la Investigación (OMI)  Uruguay 725, Ground Floor  (C1015ABO) Ciudad Autónoma de Buenos Aires  ARGENTINA  Chairperson: Dr. Marcelo Radisic |
| 705 | Comité de Revision Interna  Hospital Privado Centro Médico de Cordoba  Naciones Unidas 346  (5016) Córdoba  Córdoba  ARGENTINA  Chairperson: Dr. Sergio Metrebián |
| 706 | See above; Comité Independiente de Ética para Ensayos en Farmacología Clínica;  Comité de Docencia e Investigación del Sanatorio Parque  Boulevard Oroño 860  (2000) Rosario  Sante Fe  ARGENTINA  Chairperson: Dr. Carlos Lovesio |
| 707 | See above; Comité Independiente de Ética para Ensayos en Farmacología Clínica;  Comité de Ética en Investigación  Hospital Sirio Libanes  Campana 4658  (C1419AHN) Ciudad Autónoma de Buenos Aires  ARGENTINA  Chairperson: Dr. Roberto Cataldi Amatriain;  Comité de Docencia e Investigación  Hospital Sirio Libanes  Campana 4658  (C1419AHN) Ciudad Autónoma de Buenos Aires  ARGENTINA  Chairperson: Dr. Gustavo Frechtel |
| 708 | Comité de Ética Independiente;  Centro de Investigaciones Reumatológicas  Las Piedras 108  (T4000BRD) San Miguel de Tucumán  Tucumán  ARGENTINA  Chairperson: Dra. Gabriela Eva Maria Perez |
| 710 | See above; Comité Independiente de Ética para Ensayos en Farmacología Clínica;  Comité de Docencia e Investigación del Centro Polivalente de Asístencia e Investigación Clínica CER San Juan  Lapride 568 Este  (5400) San Juan  San Juan  ARGENTINA  Chairperson: Dr. Luis Angel Castro |
| Central IEC  Brazil | Conselho Nacional de Ética em Pesquisa – CONEP  Esplanada dos Ministérios  Bloco G, Anexo B – sala 436 b  Brasilia DF  Cep: 70.058-900  BRAZIL  Chairperson: Dr. Gyselle Saddi Tannous |
| 750 | See above; Conselho Nacional de Ética em Pesquisa – CONEP;  Comité de Ética em Pesquisa em Seres Humanos  Centro de Estudos Superiores Positivo – UNICENP/PR  Rua Pedro Viriato Parigot de Souza, 5300  Curítiba PR  Cep: 81280-330  BRAZIL  Chairperson: Dr. Maria Fernanda Torres |
| 753 | See above; Conselho Nacional de Ética em Pesquisa – CONEP;  Comité de Ética em Pesquisa da Pontifícia Universidade Católica do Rio Grande do Sul – PUC/RS  Av Ipiranga, 6690  3o andar  Porto Alegre RS  Cep: 90610-000  BRAZIL  Chairperson: Dr. Rodolfo Herberto Schneider |
| 757 | See above; Conselho Nacional de Ética em Pesquisa – CONEP;  Comité de Ética em Pesquisa em Seres Humanos das Clínicas da Universidade Federal de Goiás/GO  Primeíra Venida, s/n, Setor Leste Universitário  2^o^ andar – UPC – Unidade de Pesquisa Clínica  Goiâna GO  Cep: 74605-050  BRAZIL  Chairperson: Dr. João Carlos da Rocha Medrado |
| 760 | See above; Conselho Nacional de Ética em Pesquisa – CONEP;  Comité de Ética em Pesquisa – UNICAMP  Rua Tessália Vieira de Camargo, 126  Campinas SP  Cep: 13083-887  BRAZIL  Chairperson: Dr. Carlos Eduardo Steiner |
| 761 | See above; Conselho Nacional de Ética em Pesquisa – CONEP;  Comité de Ética em Pesquisa do Hospital Geral de Goiâna  Av. Anhanguera, 6479 – Setor Oeste  Goiâna GO  Cep: 74110-010  BRAZIL  Chairperson: Dr. Fábio Péclat do Santos |
| 801 | Comité de Ética de la Facultad de Medicina de la Universidad Autónoma de Nuevo León (UANL). y Hospital Universitario “Dr. José Eleuterio González”  Av. Francisco I. Madero y Av. Dr. Aguirre Pequeño s/n  Col. Mitras Centro  C.P. 64460, Monterrey, Nuevo León  MEXICO  Chairperson: Dr. José Gerardo Garza Leal |
| 802 | Comité de Ética en Investigación del Hospital Inovamed  Calle Cuauhtémoc #305, Col. Lomas de la Selva  C.P. 62270, Cuernavaca Morelos  MEXICO  Chairperson: Dr. Bernardo J. Rubio Cano |
| 803 | Comité Bioético para la Investigación Clinica, S.C.  Puebla # 422-4, Col. Roma Sur, Del . Cuahtémoc  C.P. 06700, México, D.F.  MÉXICO  Chairperson: Biol. Celia Ovadia Savariego |
| Central IEC  Canada | Quorum Review Insitutional Review Board  1601 Fifth Avenue, Suite 100  Seattle, WA 98101  UNITED STATES  Chairperson: David B. Kelley, MD |
| Central IEC  Canada | University Health Network Research Ethics Board  Hydro Building, 10th Floor, Suite 1056  700 University Avenue  Toronto, ON M5G 1Z5  CANADA  Chairperson: Karen McRae, MD |
| 900 | See above; Quorum Review Institutional Review Board |
| 901 | Human Investigation Committee  2nd Floor, Eastern Trust Building  95 Bonavenutre Avenue  St. John’s, NL A1B 2X5  CANADA  Chairpersons: John Harnett, MD (co-chair) and Fern Brunger, MD (co-chair) |
| 902 | See above; Quorum Review Institutional Review Board |
| 903 | See above; Quorum Review Institutional Review Board |
| 904 | See above; University Health Network Research Ethics Board |
| 905 | See above; Quorum Review Institutional Review Board |
| 907 | See above; Quorum Review Institutional Review Board |
| 908 | Mount Sinai Hospital Research Ethics Board  600 University Avenue  Toronto, ON M5X 1G5  CANADA  Chairperson: Ron Heslegrave, MD |
| 910 | See above; Quorum Review Institutional Review Board |
| Central IEC  United States | Quorum Review Institutional Review Board  1601 Fifth Avenue, Suite 100  Seattle, WA 98101  UNITED STATES  Chairperson: David B. Kelley, MD |
| Central IEC  United States | Western Institutional Review Board  3535 Seventh Avenue SW  Olympia, WA 98502  UNITED STATES  Chairperson: Theodore D. Schultz, JD |
| 950 | See above; Quorum Review Institutional Review Board |
| 951 | See above; Quorum Review Institutional Review Board |
| 952 | See above; Quorum Review Institutional Review Board |
| 953 | See above; Quorum Review Institutional Review Board |
| 954 | See above; Quorum Review Institutional Review Board |
| 957 | See above; Quorum Review Institutional Review Board |
| 958 | See above; Quorum Review Institutional Review Board |
| 959 | See above; Quorum Review Institutional Review Board |
| 960 | See above; Quorum Review Institutional Review Board |
| 961 | See above; Quorum Review Institutional Review Board |
| 962 | See above; Quorum Review Institutional Review Board |
| 963 | See above; Quorum Review Institutional Review Board |
| 964 | See above; Quorum Review Institutional Review Board |
| 965 | See above; Quorum Review Institutional Review Board |
| 966 | See above; Quorum Review Institutional Review Board |
| 967 | See above; Quorum Review Institutional Review Board |
| 968 | See above; Western Institutional Review Board |
| 969 | See above; Quorum Review Institutional Review Board |
| 970 | See above; Quorum Review Institutional Review Board |
| 971 | See above; Quorum Review Institutional Review Board |
| 972 | See above; Quorum Review Institutional Review Board |
| 973 | Cedars Sinai Medical Center Institutional Review Board  8383 Wilshire Boulevard, Suite 742  Beverly Hills, CA 90211  UNITED STATES  Chairperson: Stephen Lim, MD |
| 974 | University of California, San Diego Human Research Protection Program  8950 Villa La Jolla Drive, Suite A 208  La Jolla, CA 92037  UNITED STATES  Chairperson: Michael Caligiuri, PhD |
| 975 | See above; Quorum Review Institutional Review Board |
| 976 | MetroHealth Medical Center Institutional Review Board  2500 MetroHealth Drive, Room 103 Rammelkamp  Cleveland, OH 44109  UNITED STATES  Chairperson: David Kuentz, DO, MBA |
| 978 | See above; Quorum Review Institutional Review Board |
| 979 | See above; Western Institutional Review Board |
| 982 | Oregon Health & Science University Institutional Review Board  Mail Code L1 06, 3181 SW Sam Jackson Park Road  Portland, OR 97239  UNITED STATES  Chairperson: Susan B. Bankowski, MS, JD |
| 984 | See above; Quorum Review Institutional Review Board |
| 985 | See above; Quorum Review Institutional Review Board |
