# Supplementary material for: Discriminant validity, responsiveness and reliability of the arthritis-specific Work Productivity Survey assessing workplace and household productivity in patients with psoriatic arthritis
Source: Arthritis Res Ther. 2014 Jul 4;16(4):R140. doi: 10.1186/ar4602 (PMC4226958; doi:10.1186/ar4602)
Supplement: Additional file 2 — is a table presenting details of the questionnaires assessed in the article. [file ar4602-S2.docx]

| **Questionnaire** | **Description** | **References** |
| --- | --- | --- |
| HAQ-DI | The HAQ-DI is a patient-reported questionnaire that provides an assessment of the impact of the disease on ability to perform activities of daily living. HAQ -DI scores of 0 to 1 generally represent mild to moderate functional difficulty, 1 to 2 represent moderate to severe functional difficulty, and 2 to 3 indicate severe to very severe limitations of physical function or disability. In this study, the interpretation of within-patient changes used for the HAQ-DI scores was based on a minimum clinically important difference (MCID) of 0.3 points; this threshold was used to define responders.  The HAQ-DI was originally designed for adult arthritis patients, but has been used in a wide range of research settings and in other populations experiencing similar symptoms and impacts. The HAQ-DI contains 20 items divided into 8 domains measuring dressing and grooming, arising, eating, walking, hygiene, reach, grip, and common daily activities. Patients are required to indicate the degree of difficulty they have experienced in each item in the past week on a 4-point scale that ranges from 0 (without difficulty) to 3 (unable to do). The highest score in each domain is then summed (0 to 24) and divided by the number of domains scored to give a disability index that ranges from 0 to 3. | [[1-3](#_ENREF_1)] |
| DAS28(CRP) | The DAS28(CRP) is a composite score measuring disease activity, with lower scores indicating lower disease activity. The DAS28(CRP) is a composite score derived from the tender joint count (TJC), swollen joint count (SJC), C-reactive protein (CRP; mg/L), and Patient’s Global Assessment of Disease Activity (PtGADA, visual analog scale [VAS]) [[4](#_ENREF_4)]. The weighted sum of these components gives the DAS28(CRP), with lower scores indicating lower disease activity | [[4](#_ENREF_4)] |
| PASI | The PASI is a measure of severity of psoriatic skin involvement. The PASI ranges from 0 (no disease) to 72 (maximal disease). The PASI75 response is based on at least 75% improvement from baseline in the PASI score. In RAPID-PsA, PASI and PASI75 response were assessed in the subgroup of patients with psoriasis involving at least 3% of body surface area at baseline.  The PASI score is calculated by dividing the body into 4 sections (head [10% of a person's skin]; arms [20%]; trunk [30%]; legs [40%]), which are each scored individually and then combined. For each section, the percent of area of skin affected is estimated and then transformed into a grade from 0 to 6. Within each section, the severity is estimated by 3 clinical signs: redness, thickness and scale and graded from 0 to 4 (0=none and 4=severe). | [[5](#_ENREF_5), [6](#_ENREF_6)] |
| PsARC | A PsARC response is defined as at least a 30% improvement of tender joint count or swollen joint count, as well as a 1-point improvement on a 5-point scale in the PtGADA and the PhGADA, and no worsening of any of these scores.  The PsARC was based on tender (68 joints) and swollen (66 joints) joint counts, the Patient’s Global Assessment of Disease Activity (PtGADA, Likert scale 1-5) and the Physician’s Global Assessment of Disease Activity (PhGADA, Likert scale 1-5). The PsARC response was defined as improvement from baseline in at least 2 out of 4 measures (PtGADA, PhGADA, SJC, and TJC) 1 of which must be TJC or SJC, and no worsening from baseline in any of the 4 measures. Improvement or worsening in patient self-assessment and physician assessment was defined as decrease or increase by 1 category on the Likert scale. Joint tenderness and joint swelling improvement were defined as a decrease by ≥30% in count, and worsening as an increase by ≥30% in count. | [[7](#_ENREF_7)] |
| PsAQoL | The PsAQoL questionnaire was developed by McKenna et al. as a quality of life measure specific to adult patients with PsA. The PsAQoL global score ranges from 0 to 20 with a lower score indicating a better HRQoL.  The PsAQoL is a 20-item questionnaire with all response options as true/not true. The recall period for this instrument is the current time period. The PsAQoL global score is the sum of the answers to the 20 items each of them being scores of 0 or 1, and ranges from 0 to 20 with a lower score indicating a better HRQoL. | [[8](#_ENREF_8)] |
| DLQI | The DLQI, developed in 1994, was the first dermatology-specific quality of life instrument. The DLQI score ranges from 0 to 30, where higher scores represent greater impairment on the quality of life.  The DLQI is a simple 10-question validated questionnaire that has been used in 33 different skin conditions. The DLQI was originally developed as a brief questionnaire for routine clinical use to assess the limitations related to the impact of skin disease and has been shown to be responsive to clinical changes in a study of dermatology. The DLQI was calculated by adding the score of each question and ranges from 0 to 30, where higher scores represent greater impairment on the quality of life. | [[9](#_ENREF_9)] |
| SF-36 | The SF-36 is a widely used generic HRQoL instrument that evaluates eight health domains: physical functioning, role physical, bodily pain, general health, vitality, social functioning, role emotional, and mental health. The eight domains are summarized in two component summaries: the Physical Component Summary (PCS) and the Mental Component Summary (MCS). Scores for the SF-36 range between 0 and 100, with higher scores indicating a better HRQoL. | [[10](#_ENREF_10)] |
| EQ-5D | The EQ-5D questionnaire is comprised of a 5-item health status measure and a VAS scale. Each of the 5 dimensions is divided into 3 levels: no problem, some or moderate problems, and extreme problems and is scored as 1, 2, and 3, respectively. The EQ-5D VAS records the respondent’s self-rated health status on a vertical 20 cm scale, graduated from 0 to 100 (0=worst imaginable health status, 100=best imaginable health status). | [[11](#_ENREF_11), [12](#_ENREF_12)] |
| ACR20/50/70 | The ACR 20/50/70 response assesses the treatment of symptoms and signs in patients with active PsA. A patient is defined as an ACR 20/50/70 responder if there is an improvement (that is, reduction) of at least 20%/50%/70%, respectively, from baseline in the TJC, SJC and at least three of the five core set measures (Patient’s Assessment of Arthritis Pain (PtAAP) [VAS], PtGADA [VAS], PhGADA [VAS], an acute-phase reactant [CRP], and physical functioning based on the HAQ-DI). | [[13](#_ENREF_13)] |

**References**

1. Fries JF, Spitz P, Kraines RG, Holman HR: **Measurement of patient outcome in arthritis**. *Arthritis and rheumatism* 1980, **23**(2):137-145.

2. Bruce B, Fries JF: **The Health Assessment Questionnaire (HAQ)**. *Clinical and experimental rheumatology* 2005, **23**(5 Suppl 39):S14-18.

3. Mease PJ, Ganguly R, Wanke L, Yu E, Singh A: **How much improvement in functional status is considered important by patients with active psoriatic arthritis: applying the Outcome Measures in Rhuematoid Arthritis Clinical Trials (OMERACT) group guidelines [abstract]**. *Annals of the rheumatic diseases* 2004, **63**(Suppl 1):SAT0015.

4. Wells G, Becker JC, Teng J, Dougados M, Schiff M, Smolen J, Aletaha D, van Riel PL: **Validation of the 28-joint Disease Activity Score (DAS28) and European League Against Rheumatism response criteria based on C-reactive protein against disease progression in patients with rheumatoid arthritis, and comparison with the DAS28 based on erythrocyte sedimentation rate**. *Annals of the rheumatic diseases* 2009, **68**(6):954-960.

5. Feldman SR, Krueger GG: **Psoriasis assessment tools in clinical trials**. *Annals of the rheumatic diseases* 2005, **64 Suppl 2**:ii65-68; discussion ii69-73.

6. Fredriksson T, Pettersson U: **Severe psoriasis--oral therapy with a new retinoid**. *Dermatologica* 1978, **157**(4):238-244.

7. Clegg DO, Reda DJ, Mejias E, Cannon GW, Weisman MH, Taylor T, Budiman-Mak E, Blackburn WD, Vasey FB, Mahowald ML *et al*: **Comparison of sulfasalazine and placebo in the treatment of psoriatic arthritis. A department of veterans affairs cooperative study**. *Arthritis & Rheumatism* 1996, **39**(12):2013-2020.

8. McKenna SP, Doward LC, Whalley D, Tennant A, Emery P, Veale DJ: **Development of the PsAQoL: a quality of life instrument specific to psoriatic arthritis**. *Annals of the rheumatic diseases* 2004, **63**(2):162-169.

9. Finlay AY, Khan GK: **Dermatology Life Quality Index (DLQI)--a simple practical measure for routine clinical use**. *Clinical and experimental dermatology* 1994, **19**(3):210-216.

10. Ware JE, Jr., Sherbourne CD: **The MOS 36-item short-form health survey (SF-36). I. Conceptual framework and item selection**. *Medical care* 1992, **30**(6):473-483.

11. Brooks R: **EuroQol: the current state of play**. *Health policy* 1996, **37**(1):53-72.

12. EuroQol G: **EuroQol--a new facility for the measurement of health-related quality of life**. *Health policy* 1990, **16**(3):199-208.

13. Felson DT, Anderson JJ, Boers M, Bombardier C, Chernoff M, Fried B, Furst D, Goldsmith C, Kieszak S, Lightfoot R *et al*: **The American College of Rheumatology preliminary core set of disease activity measures for rheumatoid arthritis clinical trials. The Committee on Outcome Measures in Rheumatoid Arthritis Clinical Trials**. *Arthritis and rheumatism* 1993, **36**(6):729-740.
