# Supplementary material for: Discriminant validity, responsiveness and reliability of the arthritis-specific Work Productivity Survey assessing workplace and household productivity in patients with psoriatic arthritis
Source: Arthritis Res Ther. 2014 Jul 4;16(4):R140. doi: 10.1186/ar4602 (PMC4226958; doi:10.1186/ar4602)
Supplement: Additional file 3 — is Tables S1 and S2 presenting WPS baseline scores assessed by defined known-groups. Known-groups analysis using median scores as the cutoff for analysis. [file ar4602-S3.docx]

Table S1: WPS baseline scores assessed by defined known-groups: patient workplace productivity (RS, observed cases)

| **Instrument^a^** | **Number of days of work missed over the previous month, mean** | | **Number of days with productivity ≤50% at work over the previous month, mean** | | **Rate of arthritis interference with work productivity over previous month, mean^b^** | |
| --- | --- | --- | --- | --- | --- | --- |
|  | **Worse** | **Better** | **Worse** | **Better** | **Worse** | **Better** |
| **HAQ-DI**  (cutoff 1.13) | 2.85^#^  n=110 | 1.36  n=133 | 6.83**  n=110 | 2.98  n=133 | 5.19**  n=109 | 3.25  n=133 |
| **DAS28(CRP)**  (cutoff 4.83) | 2.75^#^  n=121 | 1.33  n=122 | 5.93*  n=121 | 3.52  n=122 | 4.72**  n=120 | 3.54  n=122 |
| **SF-36 PCS**  (cutoff 35.38) | 2.67  n=121 | 1.42  n=121 | 6.26**  n=121 | 3.21  n=121 | 5.05  n=120 | 3.24  n=121 |
| **SF-36 MCS**  (cutoff 44.29) | 2.53  n=121 | 1.56  n=121 | 5.79^#^  n=121 | 3.69  n=121 | 4.69*  n=120 | 3.60  n=121 |
| **PsAQoL**  (cutoff 10.00) | 3.74**  n=107 | 0.70  n=136 | 6.58**  n=107 | 3.26  n=136 | 5.09**  n=106 | 3.37  n=136 |
| **PASI**  (cutoff 7.00) | 1.96  n=76 | 2.44  n=78 | 4.80  n=76 | 3.53  n=78 | 3.58  n=76 | 4.27  n=78 |
| ^a^Cutoff point represents the median baseline scores: “Worse” state defined for each individual measure as: DAS28(CRP) score ≥median; HAQ-DI ≥median; PsAQoL ≥median; SF-36 MCS ≤median; SF-36 PCS ≤median; PASI ≥median; “Better” state defined for each individual measure as: DAS28(CRP) score ≤median; HAQ-DI ≤median; PsAQoL ≤median; SF-36 MCS ≥median; SF-36 PCS ≥median; PsAQoL ≤median; ^b^WPS Q4 is a 0 to 10 scale, where 0=no interference and 10=complete interference; **p-value≤0.001; *p-value≤0.01; ^#^p-value≤0.05; nonparametric bootstrap-t method with a variance stabilizing transformation; 10,000 replications. | | | | | | |

Table S2: WPS baseline scores assessed by defined known-groups: household productivity and daily activities (RS, observed cases)

| **Instrument^a^** | **Number of days of household work missed over the previous month, mean** | | **Number of days with household productivity ≤50% at work over the previous month, mean** | | **Number of days of missed family, social, or leisure activities over the previous month, mean** | | **Number of days with outside help over the previous month, mean** | | **Rate of arthritis interference with household work productivity over previous month, mean^b^** | |
| --- | --- | --- | --- | --- | --- | --- | --- | --- | --- | --- |
|  | **Worse** | **Better** | **Worse** | **Better** | **Worse** | **Better** | **Worse** | **Better** | **Worse** | **Better** |
| **HAQ-DI**  (cutoff 1.38) | 9.37**  n=178 | 2.77  n=228 | 10.53**  n=178 | 5.28  n=228 | 6.06**  n=178 | 1.90  n=228 | 4.35**  n=178 | 0.96  n=228 | 6.36**  n=178 | 3.90  n=228 |
| **DAS28(CRP)**  (cutoff 4.92) | 7.83**  n=204 | 3.48  n=202 | 9.23**  n=204 | 5.92  n=202 | 5.30**  n=204 | 2.12  n=202 | 3.70**  n=204 | 1.18  n=202 | 5.75**  n=204 | 4.20  n=202 |
| **SF-36 PCS**  (cutoff 32.91) | 8.58**  n=202 | 2.72  n=198 | 9.80**  n=202 | 5.37  n=198 | 5.54**  n=202 | 1.97  n=198 | 3.56**  n=202 | 1.36  n=198 | 6.03**  n=202 | 3.97  n=198 |
| **SF-36 MCS**  (cutoff 41.75) | 8.07**  n=201 | 3.26  n=199 | 9.47**  n=201 | 5.73  n=199 | 6.21**  n=201 | 1.32  n=199 | 3.65**  n=201 | 1.28  n=199 | 5.80**  n=201 | 4.22  n=199 |
| **PsAQoL**  (cutoff 12.00) | 8.64**  n=182 | 3.20  n=223 | 10.31**  n=182 | 5.32  n=223 | 6.94**  n=182 | 1.11  n=223 | 4.52**  n=182 | 0.77  n=223 | 6.16**  n=182 | 4.00  n=223 |
| **PASI**  (cutoff 7.20) | 4.89  n=123 | 6.72  n=127 | 7.79  n=123 | 6.39  n=127 | 3.45  n=123 | 4.24  n=127 | 2.63  n=123 | 2.13  n=127 | 4.52  n=123 | 5.23  n=127 |
| ^a^Cutoff point represents the median baseline scores: “Worse” state defined for each individual measure as: DAS28(CRP) score ≥median; HAQ-DI ≥median; PsAQoL ≥median; SF-36 MCS ≤median; SF-36 PCS ≤median; PASI ≥median; “Better” state defined for each individual measure as: DAS28(CRP) score ≤median; HAQ-DI ≤median; PsAQoL ≤median; SF-36 MCS ≥median; SF-36 PCS ≥median; PsAQoL ≤median; ^b^WPS Q4 is a 0 to 10 scale, where 0=no interference and 10=complete interference; **p-value≤0.001; *p-value≤0.01; nonparametric bootstrap-t method with a variance stabilizing transformation; 10,000 replications. | | | | | | | | | | |
